# The association between resource availability and knowledge, attitudes, and practice about protective ventilation among ICU directors – A nationwide cross-sectional survey

**DOI:** 10.1371/journal.pone.0355354

**Published:** 2026-08-14

**Authors:** Sângia Feucht Freire, Iara Sayuri Shimizu, Cristhiano A. S. Lima, Neill K. J. Adhikari, Alfred Papali, Juliana Carvalho Ferreira

**Affiliations:** 1 Divisao de Pneumologia, Instituto do Coracao (InCor), Hospital das Clinicas HCFMUSP, Faculdade de Medicina, Universidade de Sao Paulo, Sao Paulo, Sao Paulo, Brazil; 2 Coordenacao de Fisioterapia, Centro de Ciencias da Saude, Universidade Estadual do Piaui (UESPI), Teresina, Piaui, Brazil; 3 Department of Critical Care Medicine, Sunnybrook Health Sciences Centre, and Interdepartmental Division of Critical Care Medicine, University of Toronto, Toronto, Canada; 4 Division of Pulmonary and Critical Care Medicine, Atrium Health-Wake Forest University School of Medicine, Charlotte, North Carolina, United States of America; Government Villupuram Medical College and Hospital, INDIA

## Abstract

**Objectives:**

To assess the association between geographic region and availability of ICU resources in Brazil, as well as the relationship between resource availability and KAP for protective ventilation.

**Methods:**

We conducted a cross-sectional study of ICU directors using a validated online survey that included 29 items about ICU characteristics and the availability of 38 resources, and 27 KAP items. We used a Likert scale to score KAP items and calculated a KAP score as the sum of the points, standardized on a scale of 0–100. We used multiple linear regression to assess associations between geographic regions, resources, and KAP.

**Results:**

We included 255 ICUs, 12% of the country’s ICUs, in a geographically representative distribution of all regions in Brazil. The median of resources available was 34 (interquartile range [IQR] 33–36) out of 38, with statistically significant inequalities among regions, p < 0.001 and 178 (70%) of participants reported an increase in resources available after the COVID-19 pandemic. The median KAP score was 83 (IQR 77–90) and there was a significant association between a greater number of resources and higher KAP (p = 0.031).

**Conclusion:**

In this study including a representative sample of ICUs in Brazil, we identified regional inequalities in the availability of ICU resources. KAP scores regarding protective ventilation were generally high but varied across regions. Although greater resource availability was associated with higher KAP scores, the magnitude of this association was modest, suggesting that factors beyond resource availability may also contribute to differences in knowledge, attitudes, and practices related to protective ventilation.

## Introduction

The burden of critical illness is disproportionately high in low-and-middle-income countries (LMICs) [1,2]. Previous studies have found that mortality rates for adults with sepsis [2–4] and acute respiratory distress syndrome (ARDS) [5–7] are higher in resource-limited settings compared to resource-rich settings. This disparity can be attributed to several factors, including scarcity of intensive care unit (ICU) beds and other resources, limited access to healthcare services, lack of early recognition of critical illness, and lack of implementation of evidence-based treatments [1,8].

Protective mechanical ventilation, defined as the use of low tidal volumes and limited plateau pressures, is associated with improved survival [9] and is recommended for patients with ARDS [10]. However, several studies show that its implementation is low, even in high resource settings [11]. Several factors contribute to the challenges of implementation of protective ventilation strategies in LMICs. One significant barrier is the limited availability of resources, including both equipment, disposable materials, and specialized healthcare professionals trained in recognizing and managing ARDS [12].

Knowledge deficits and negative attitudes towards the use of protective ventilation can lead to low adherence to protective ventilation and impact patient outcome [13]. Measuring knowledge, attitudes and practices (KAP) towards protective ventilation and understanding the barriers to its adoption is important for promoting implementation of evidence-based ventilatory strategies and improving outcomes for ARDS patients [13,14]. In addition, mapping the availability of resources and its relationship with protective ventilation KAP is crucial for promoting rational resource allocation and developing health policies to improve adherence to protective ventilation and other evidence-based treatments, with the final goal of improving patient outcomes. This study aimed to assess the association between geographic region and availability of ICU resources in Brazil, as well as the relationship between resource availability and KAP related to protective ventilation for patients with ARDS. We hypothesized that there would be regional disparities in resource availability, reflecting well known regional disparities, rooted in historical, systemic disparities in economic development and infrastructure. We also hypothesized that lack of resources would be associated with lower KAP regarding protective ventilation as limited resources may be associated with reduced staffing, access to training, availability of treatment protocols and quality improvement initiatives, with inincreased workload and implementation barriers, ultimately affectting the knowledge, acceptance, and consistent application of protective ventilation practices.

## Materials and methods

### Design, setting and participants

This was a cross-sectional study, using official and publicly available databases from the Ministry of Health in Brazil and validated questionnaires. The study was approved by the ethics committee of Hospital das Clínicas da Faculdade de Medicina da Universidade de São Paulo (approval number 3241885) and informed consent was waived due to the observational nature of the study. The study was conducted with the partnership of the Brazilian Critical Care Medicine Association (Associação de Medicina Intensiva Brasileira, AMIB) and the Brazilian Research in Intensive Care Network (BRICnet).

Participants were medical directors of adult ICUs, including coronary care units and burns ICUs. Exclusion criteria were refusal to participate, pediatric ICUs or intermediate care units, as classified by the Ministry of Health. In Brazil, federal regulations require that ICUs have a board-certified intensivist as medical director, and daily care of patients be provided by a physician for each 10 ICU beds, one chief nurse for each five beds, one assistant nurse for each two beds, and one physiotherapist for each 10 ICU beds. In Brazil, there are no respiratory therapists, but physiotherapists who specialize in respiratory care. Therefore, mechanical ventilation adjustments are usually made by physicians and physiotherapists.

Data collection lasted 14 months (May 1^st^ 2021 to June 30 2022). The questionnaire link was sent by e-mail to a mailing list of all AMIB and BRICnet members, inviting those who were medical directors to participate, with three weekly reminders, and by social media and instant messaging groups, using a snowball strategy for recruitment.

We excluded incomplete responses in which the answers to knowledge, attitudes and practices (KAP) or the availability of resources items were missing.

### Survey development

We used previously validated questionnaires to create our survey as a self-administered, online survey, housed in a secure Redcap platform [15,16].

The final version of the survey had 56 items. The first 29 items were based on a validated self-administered questionnaire for health care providers developed to estimate the availability of resources for the management of critically ill patients in resource-limited settings [17]. The items addressed hospital characteristics and the availability of 38 resources. Of the 38 resources, 10 were considered essential, based on a recommendation of the European Society of Intensive Care Medicine (ESICM) Global Intensive Care working group [3]. In addition, we included one question from a Brazilian multicenter study [18] regarding multidisciplinary rounds and two questions about the impact of COVID-19 pandemic on hospital resources and the practice of protective ventilation.

The second part of the survey reproduced a previously published questionnaire evaluating KAP in the use of protective ventilation in patients with ARDS and the barriers to implementation [13]. There were 18 KAP items, 10 assessed attitude, three assessed self-reported practices, and five assessed knowledge. In addition, there were five items assessing barriers and four knowledge items structured as multiple-choice questions [13,14].

We added two additional steps: translation from English to Portuguese, with back-translation to check the overall quality of the translation and retain the original meaning; and pilot testing of the survey with 22 Brazilian ICU medical directors, selected by convenience sampling. These steps led to adjustments on formatting of the questionnaire and small adjustments to the translation.

### Survey scoring

To quantify available resources, we assigned one point for each resource marked as available by respondents, and calculated the sum of these points, ranging from 0 to 38, and as a percentage of all 38 items. We also calculated the sum of the points for the 10 essential resources, and the respective percentage.

For the 18 subjective KAP items, we used a 6-point Likert scale, with the following response options: strongly agree, agree, neutral, disagree, strongly disagree, do not know. The statements alternated between positive and negative wording to avoid response set bias. Positively worded items were scored from 5 to 0 points, where 5 was attributed to strongly agree and 1 was attributed to strongly disagree. We used reverse scores for negatively worded items, i.e., 5 was attributed to strongly disagree and 1 attributed to strongly agree. The “Do not know” option scored 0 points for all items. A knowledge, attitude and practice score (KAP score) were calculated as the sum of the points of the 18 subjective knowledge, attitude and practice items, and standardized into a uniform scale ranging from 0 to 100, with higher scores indicating greater KAP. There is no universally accepted cut off for KAP scores, so we used the commonly adopted Bloom’s cut-off points, as follows: 80%–100% (high/good KAP), 60%–79% (moderate KAP) and less than 60% (low/poor KAP).

The four objective knowledge items were scored as correct or incorrect, and each item scored 1 point. Scores ranged from 0 to 4, with a higher score indicating a higher knowledge level, and scores equal or greater than 3 were considered as high knowledge.

The five perceived barriers were scored with the same 6-point Likert scale as the KAP items, with higher scores meaning greater perceived barriers.

### National databases

To map the availability of ICU beds in the country, we used an official Brazilian national database of ICUs, updated in 2018, which describes the number of ICUs and ICU beds for the entire country by each of five regions/provinces and 27 states [19]. The database classifies ICUs as high or medium level (according to human and technological resources, using standard government criteria used to allocate financial resources). Population data for each region of the country was obtained from the closest population census, which was completed in 2015 [20].

### Statistical analysis

Continuous variables were described as median and interquartile ranges (IQR) and categorical variables are described as count and percentages. We used linear regression to estimate the association of ICU characteristics and ICU resources with KAP. Multivariable models were used to adjust for confounding. The multivariable models were based on conceptual causal diagrams in the form of directed acyclic graphs (DAG) including relevant covariates, such as number of hospital beds, type of hospital funding, staffing, ICU participation in clinical research, use of daily multidisciplinary rounds, among others, and tested the association between region of the country and resources (S1 Fig), and resources and KAP (S2 Fig).

Statistical analysis was performed using the R statistical package, version 4.0.3 (The R Foundation for Statistical Computing, Vienna, Austria) and statistical significance was set at p < 0.05.

The reporting of study results was written following the STrengthening the Reporting of OBservational studies in Epidemiology (STROBE) reporting guidelines [21].

## Results

Of the approximately 3700 AMIB members invited by e-mail and approximately 600 social media invitations, we received 264 responses. We are unable to estimate the response rate because the mailing database included all active AMIB members, but most members are not ICU medical directors, while some of the 2085 medical directors of the ICUs listed in the national database may not be active members of the society. We excluded 7 responses in which response to the KAP items or resource availability items were missing and 2 responses from pediatric ICU medical directors.

The final sample represents 255 ICUs distributed in all states of Brazil corresponding to 12% of all ICUs listed in the database in 2018. The distribution of ICUs per region in our sample was representative of the ICU distribution in Brazil, with a slight underrepresentation of the northeast region (20% in the national database vs 15% in our sample). Most of the hospitals were public (n = 113, 44%), with a median of 20 (10–30) ICU beds, and the vast majority had a physician present 24/7. Other characteristics of the ICUs are described in Table 1.

**Table 1:** Characteristics of sampled ICUs (n = 255).

ICU location per region in Brazil – n (%)	
North	23 (9%)
Northeast	39 (15%)
Central west	14 (6%)
Southeast	134 (52%)
South	45 (18%)
**Location in the State** – n (%)	
State capital	149 (58%)
Other cities	106 (42%)
**Hospital funding** – n (%)	
Public	113 (44%)
Private	93 (37%)
Philanthropic	34 (13%)
Military	3 (1%)
Academic	12 (5%)
**Number of hospital beds**	
< 50	16 (6%)
50–100	37 (15%)
101–500	177 (69%)
501–1000	21 (8%)
1001–2000	3 (1%)
> 2000	1 (0%)
**ICU type**	
Medical	73 (29%)
Surgical	12 (5%)
Mixed	153 (60%)
Other	17 (6%)
**Number of ICU beds** – Median (IQR)	20 (10–30)
**ICU bed occupancy**	
0 - 25%	4 (2%)
26 - 50%	8 (3%)
51 - 75%	48 (19%)
76 - 100%	195 (77%)
**Physician present 24/7 – n (%)**	251 (98%)
**ICU bed per nurse**– Median (IQR)	10 (7–25)
**Percent of beds with MV capability** – Median (IQR)	100 (100–100)
**ICU participation in clinical research** – n (%)	107 (42%)
**Daily multidisciplinary rounds**	87 (34%)

ICU: intensive care unit; MV: mechanical ventilation. ICU nurse per bed and number of ICU beds missing for 2 respondents, percent of beds with MV capability missing for six respondents.

### Resources

According to national databases, Brazil had 1806 hospitals with 2085 adult ICUs in 2018, of which 97 were coronary ICUs and 47 were burn ICUs [19]. The median number of ICUs and ICU beds per state was 38 (IQR 23–85) and 562 (IQR: 249–1196), respectively. The medium number of beds per ICU was 10 (IQR 8–20). The Southeast region, which includes the richest cities in the country, had the highest number of ICU beds per inhabitant, while the North region, which includes the Amazon, had the lowest number of ICU beds per inhabitant (S1 Table).

**Fig 1:**
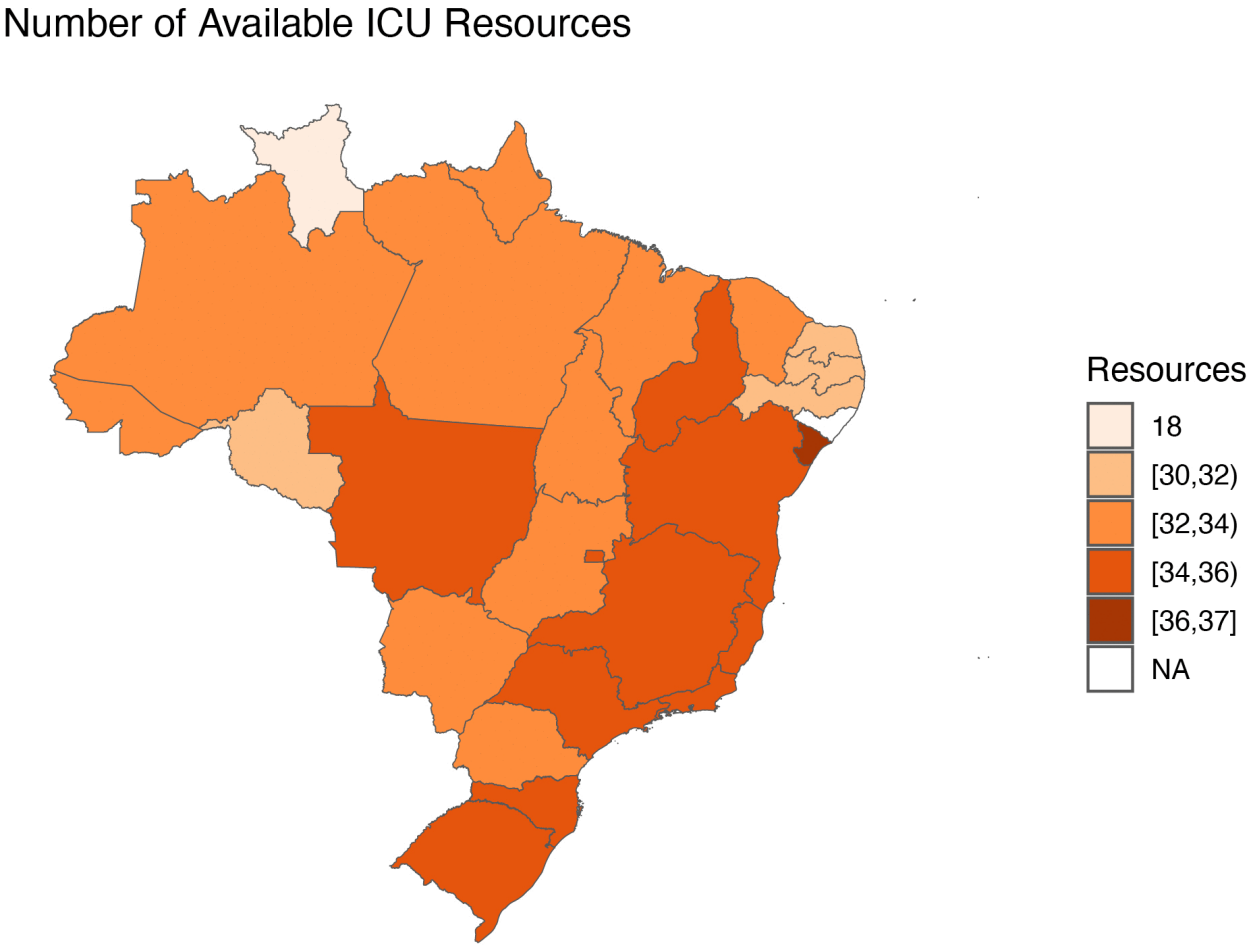
Map of Brazilian states showing the average number of ICU resources available in each state. Resource availability is represented by a color gradient ranging from light orange to dark brown, with lighter shades indicating fewer resources and darker shades indicating greater resource availability. States are grouped into five categories according to the average number of resources, with category ranges shown in the legend. One state was not represented in our sample and is shown in white.

Similarly, in our sample, a modest but significant variation in ICUs resources available in different Brazilian regions was observed. The Southeast region had a median of 35 (34−36) resources out of 38, the north region had 33 (31−34), the northeast region had 34 (31−35), the south region had 34 (33−36), and central west had 33 (33−36), with a significant association between region of the country and number of resources available, p < 0.001 (S2 and S3 Tables). Fig 1 shows a map with the distribution of resources across the country and S2 Table shows that most of the states in the North and Northeast regions are below the national median number of resources. Other factors statistically associated with more ICU resources were participation in clinical research (p = 0.016), number of hospital beds (p < 0.001), and multidisciplinary rounds (p < 0.001) (S3 Table). Most ICUs in our sample had all 10 essential resources, with oxygen (centralized system or cylinders) available in all ICUs and point-of-care ultrasound available in 239 (94%) (Table 2). The median number of essential resources was 10 (IQR 10–10) and the median number of resources available was 34 (IQR 33−36) out of 38. We also found that 178 (70%) of ICU medical directors reported an increase in available resources during or after the COVID-19 pandemic.

**Table 2:** Essential resources reported as available in the sampled ICUs.

Essential resources – n (%)	
Oxygen*	255 (100%)
Invasive ventilatory support	251 (98%)
Vital Signs monitors	250 (98%)
Glucometers	250 (98%)
Alcohol-based solution	250 (98%)
Personal protective equipment (PPE)^#^	250 (98%)
Non-invasive ventilation	247 (97%)
Back-up electrical sources	244 (96%)
Measuring blood lactate levels (hospital)	241 (95%)
Point-of-care ultrasound	239 (94%)
**Other resources – n (%)**	
Intravenous inotropes or vasopressors	253 (99%)
Intravenous antibiotics	253 (99%)
Intravenous sedation	253 (99%)
Intravenous analgesia	253 (99%)
Central venous catheters	253 (99%)
Nasogastric tubes	253 (99%)
Chest tubes	253 (99%)
Defibrillators	253 (99%)
Intravenous crystalloid fluids	252 (99%)
Intravenous drug infusion pumps	252 (99%)
ECG portable machines	251 (98%)
Renal replacement therapy	247 (97%)
Arterial blood gas analysis	247 (97%)
Packed red blood cells	246 (97%)
CT scan	245 (96%)
Portable X-Ray machine	243 (95%)
Plasma	243 (95%)
Arterial catheters	243 (95%)
Platelets	241 (95%)
Whole blood	236 (93%)
X-Ray machine (not portable)	227 (89%)
More nurses per patients than on a general ward	200 (78%)
Microbiology laboratory (on site)	195 (77%)
MRI Scan	138 (54%)
Single patient rooms	135 (53%)
Negative pressure isolation rooms	106 (42%)

*The three types of oxygen delivery systems (centralized, cylinders and concentrators) were grouped into a single resource. ^#^PPE included gloves, masks, caps, sterile gowns, sterile drapes and sterile gloves for invasive procedures. The available resources were described in descending order of availability.

### KAP towards protective ventilation

**Fig 2:**
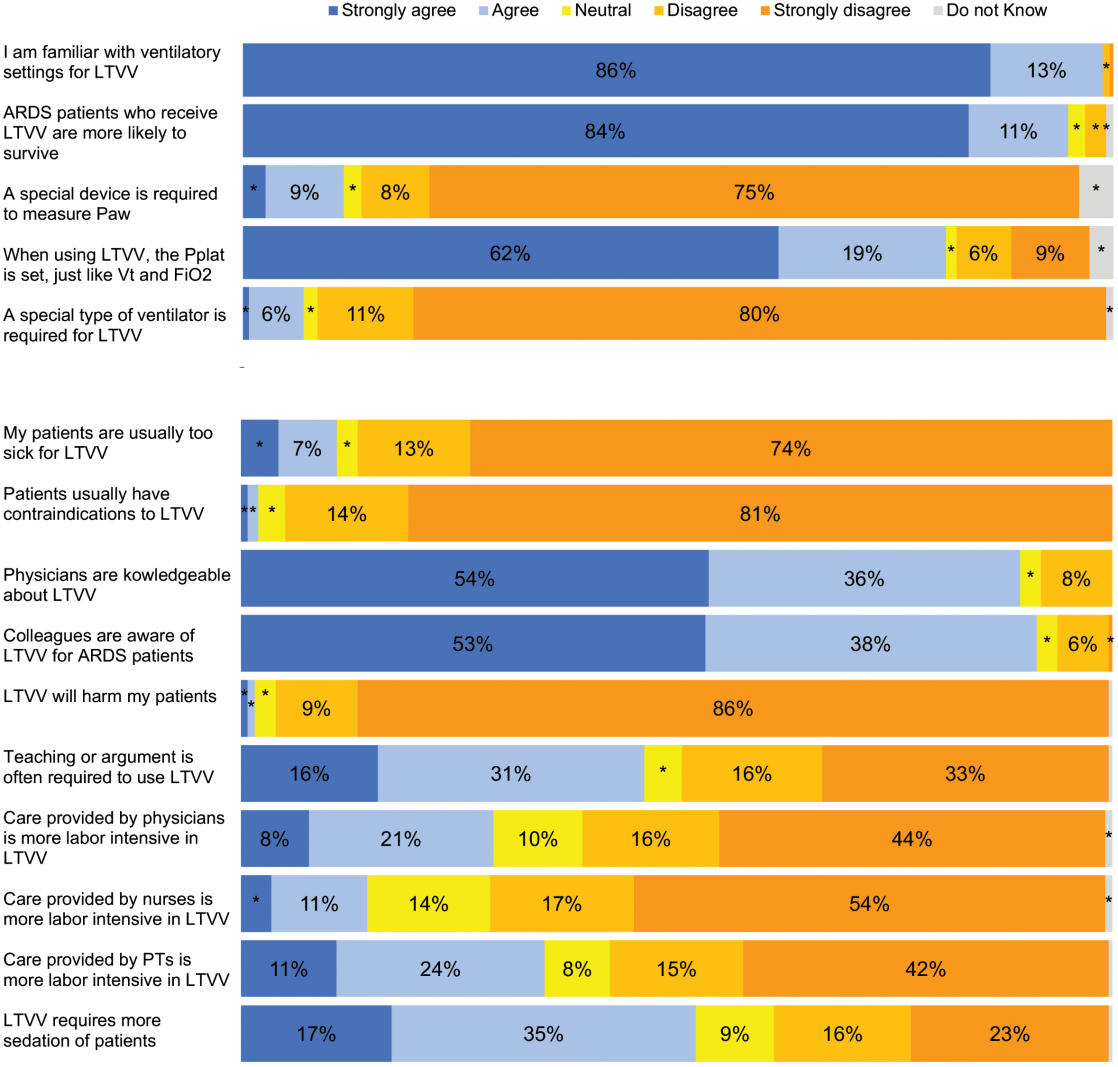
Knowledge (panel A) and Attitude (panel B) about low tidal volume ventilation. Legend: LTVV: Low Tidal Volume Ventilation, ARDS: Acute Respiratory Distress Syndrome, Paw: Airway Pressure, Vt: Tidal Volume, FiO2: Fraction of Inspired Oxygen, PTs: Physical therapists. * ≤ 5%.

**Fig 3:**
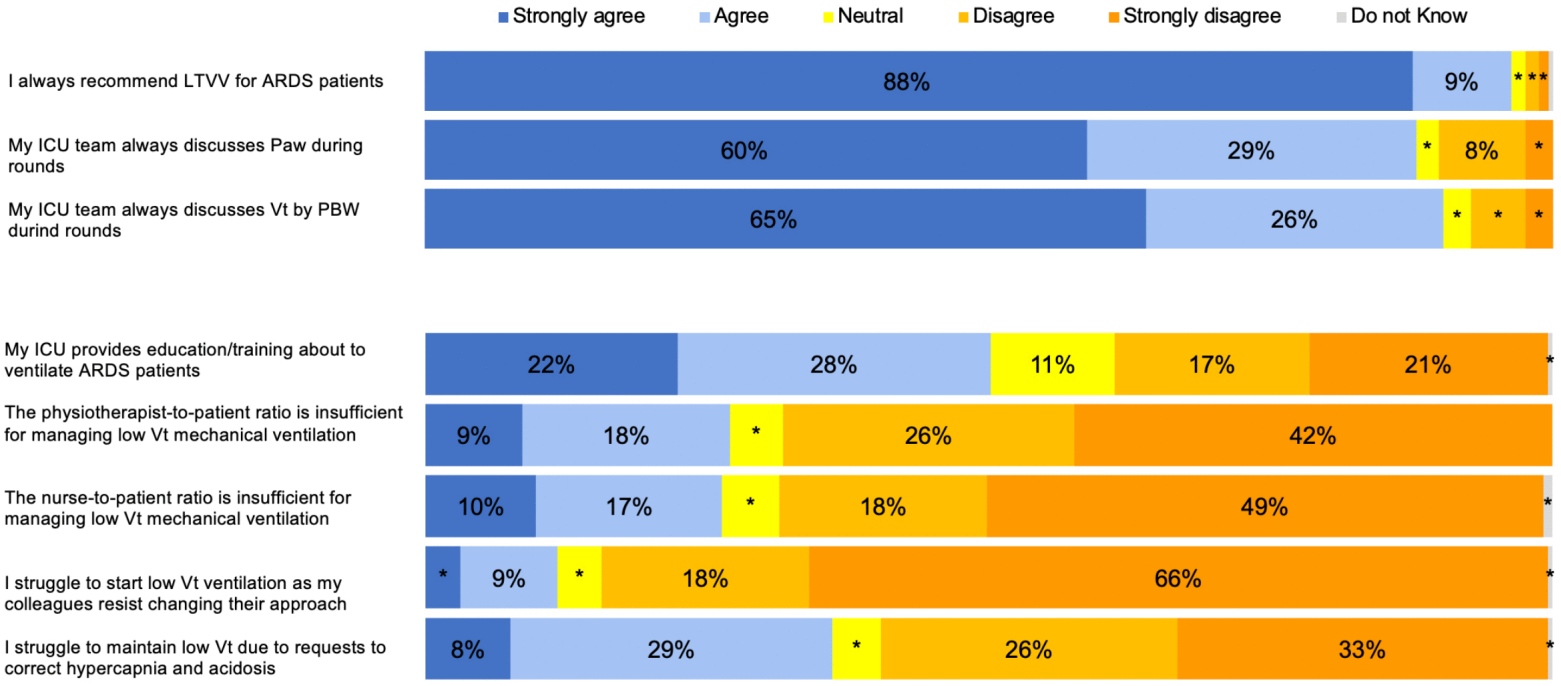
Practice (panel A) and Barriers (panel B) regarding protective ventilation. Legend: LTVV: Low Tidal Volume Ventilation, ARDS: Acute Respiratory Distress Syndrome, ICU: Intensive Care Unit, Paw: Airway Pressure, Vt: Tidal Volume, PBW: Predicted Body Weight. * ≤ 5%.

The results of the KAP section of the survey are summarized in Figs 2 and 3. The median knowledge score was 84 (76–84) out of 100, with 95% of participants agreeing that low tidal volume ventilation improves survival for ARDS patients, but 81% agreeing with the false statement that plateau pressure is set during low tidal volume ventilation (Fig 2, panel A). The median score for attitude was 82 (71–91), with more than 50% of strongly agree/agree or strongly disagree/disagree for all items, except for the item regarding the need for more sedation to implement low tidal volume ventilation (Fig 2, panel B). The median practice score was 93 (87–100) (Fig 3, panel A). The barriers median score was 44 (IQR: 32–56), with the most significant barriers being lack of training (38% disagree/strongly disagree that their ICU offer training) and difficulties to maintain low tidal volumes when other members of the team request ventilatory changes to address hypercapnia and acidosis (37% agree or strongly agree) (Fig 3, panel B).

The median overall KAP score was 83 (IQR 77–90) out of 100, which is considered high/good KAP. In univariable analyses, the region of the country, hospital funding, number of hospital beds, ICU beds per nurse, and participation in research were not significantly associated with KAP score (S4 Table). In contrast, the number of ICU resources was associated with higher KAP scores (p = 0.007), as well as 24/7 presence of a physician (p = 0.05) and multidisciplinary daily rounds (p = 0.03). In the multivariable model, adjusted for participation in research and multidisciplinary rounds, as indicated by our DAG model, only the number of available resources was statistically associated with KAP (p = 0.031) (S4 Table).

## Discussion

In this observational study of a sample of 255 ICUs from all states of Brazil, we found that the vast majority of the ICUs have all resources considered essential. However, we found significant variability of resources across different country regions, with a concentration of resources in the richest regions of the country. Participants had a high level of knowledge, attitudes, and practice towards protective ventilation, and the most important barrier to implementation of protective ventilation was lack of training. There was a significant but modest association between the number of resources available and a higher KAP score towards protective ventilation.

We surveyed 12% of the ICU medical directors in a country with more than 200 million inhabitants. The distribution of the participating ICUs was representative of the regions of the country, and most ICUs were publicly funded. In our sample, the ICU resources were associated with region of the country, with more resources in the Southeast region and less in the North region.

All states in the North region and most states in the Northeast region had a lower median number of ICU resources than the national median, although the absolute differences in resource availability were modest. Nevertheless, this pattern mirrors broader regional inequalities in critical care infrastructure in Brazil. National databases show that the number of ICU beds per capita in the North region is less than half that of the Southeast region [19], suggesting that even small differences in resource availability may reflect larger structural disparities in access to critical care.

These findings reflect a broader landscape of unequal access to critical care services in Brazil. Although the average number of ICU beds in Brazil (2.03 per 10,000 inhabitants) is considered high, regional, racial and economic inequalities in access to care are well documented [22,23]. For example, the public, universally accessible health system has 1.3 ICU beds per 10,000 inhabitants while the private sector offers 4.45 ICU beds per 10,000 beneficiaries [19]. The most recent mapping of regional distribution of ICU beds, completed in 2024, after the impact of the COVID-19 pandemic shows a similar pattern of regional inequality [24].

Mapping regional inequalities is important because health resource allocation has a direct impact on patient outcomes. During the COVID-19 pandemic, regional inequalities in ICU bed availability led to broad disparities in the countries capacity to respond to the strain imposed by COVID-19, with higher mortality rates in less resourced areas [25] and among black and economically vulnerable populations [22]. Importantly, 70% of our respondents reported that the availability of resources increased during or after the COVID-19 pandemic, although the survey did not record which resources, nor did we ask about the sustainability of this increase over time, after the pandemic was controlled.

The availability of the 10 resources considered essential was very high, with all ICUs reporting the availability of oxygen, for example. Cautious extrapolation of this finding to all Brazilian ICUs is required, since there is a risk of selection bias among respondents to the survey. Oxygen is considered an essential medicine by the World Health Organization’s (WHO) [26], but the optimal delivery system and dose are not well determined [3,27]. There are few studies about oxygen availability in LMICs, mostly focused on African countries, which show that under recognized and untreated hypoxemia are associated with high mortality [27]. During the COVID-19 pandemic, disparities in oxygen availability surfaced in many countries, including Brazil, where the lack of oxygen in the Amazon region was the marker of health care system collapse and led to record daily deaths [28], exposing the fragility of the chronically strained Brazilian health system. The availability of the 38 resources was high, with 75% of ICUs having 33 (87%) resources or more.

Our study identified structural and organizational aspects of ICUs associated with more resources. Hospital size, measured by the number of hospital beds, had a positive association with number of available resources, indicating that ICU resources are concentrated in larger hospitals. In addition, reporting of participation in clinical research and the routine of a daily multidisciplinary round were also associated with more resources. Regular multidisciplinary rounds are shown to improve patient satisfaction and safety [29] and have already been identified as associated with patient outcomes in a large multicentric study in Brazil in 2014 [18], and this association remains consistent over time [30]. For both participation in research and multidisciplinary rounds, the association with more resources may reflect more human resources or more specialized teams. Our list of 38 resources only includes one question about ICU staffing, namely if the ICU has more nurses per patient than the hospital´s general ward. Therefore, we can only speculate that ICUs with better organizational structures and more access to important technical resources also tend to have more specialized teams and a greater opportunity to leverage those assets to improve patient care and foster research.

Knowledge about protective ventilation was high, in contrast with the original publication of the KAP survey [13]. This difference may be due to the greater dissemination and awareness of guideline recommendations to use lung protective ventilation [10,31,32] in the 15 years that separated the two studies. Participants reported a high level of familiarity with protective ventilation and expressed the belief that low tidal volume ventilation improves survival outcomes for patients with ARDS. However, more than half of the participants incorrectly believed that plateau pressure was a set parameter for low tidal volume ventilation. This inconsistency suggests that participants may overestimate their knowledge regarding the specific ventilatory settings required for protective ventilation strategies.

Participants generally held positive attitudes towards protective ventilation. Most respondents disagreed with the notion that patients with ARDS typically have contraindications for low tidal volume ventilation or that low tidal volume ventilation harms ARDS patients. These positive attitudes could be attributed to the increasing dissemination of the clinical impact of protective ventilation [10] and may also reflect improved familiarity imposed by the COVID-19 pandemic [33]. Divergent attitudes were observed when participants were asked about the increased sedation requirements associated with protective ventilation compared to conventional ventilation, despite recent evidence that lighter sedation is safe and as effective as deep sedation and neuromuscular blockade in ARDS [34]. This finding may be explained by the perception that acutely ill patients require deep sedation to ensure better oxygenation and limit driving pressure [35].

Our study found a high prevalence of reported adherence to protective ventilation practices. Most participants claimed to consistently recommend protective ventilation for ARDS patients and reported discussing airway pressures and tidal volume by predicted weight during rounds. However, this self-reported adherence may not reflect actual bedside practices, as studies have shown discrepancies between stated practice and observed behaviors, with modest adherence to protective ventilation protocols for ARDS patients [11,36].

The most important barriers to the implementation of protective ventilation identified by participants were related to acidosis and hypercapnia and ICU training. Difficulties in maintaining low tidal volumes due to concerns about hypercapnia and acidosis were reported by 37% of participants despite evidence indicating that permissive hypercapnia does not increase mortality in ARDS [37]. This finding underscores the need for adequate training that includes indications, contraindications and implementation strategies. Evidence suggests that training improves adherence to low tidal volume ventilation [38–40] and may also have an impact on healthcare worker wellbeing and patient outcomes.

There was a positive and significant association between more reported ICU resources and higher KAP towards mechanical ventilation, even after adjusting to potential confounders. However, the magnitude of the association was small, and median KAP scores were high among the ICU directors in our sample. Several interpretations are possible, and our observational design does not allow causal inferences. Although we adjusted for known structural and organizational ICU characteristics, the modest effect size suggests that unmeasured factors beyond resource availability likely contribute to differences in KAP. Resource availability may reflect broader aspects of ICU organization, such as staff qualification, institutional culture, training opportunities, and quality improvement initiatives, which could also influence the adoption of evidence-based practices. Therefore, simply increasing the number of resources is unlikely, by itself, to substantially improve KAP regarding protective ventilation or other recommended practices. Instead, ICUs with fewer resources may have more organizational and educational needs, and multifaceted interventions addressing infrastructure, workforce development, and training, may be required to improve implementation of best practices [41].

### Limitations

Our study has several limitations. First, our sample may be biased towards ICUs with more resources, structure, and more academic engagement. Our sampling method using medical association mailing lists may have selected a nonrepresentative sample of Brazilian ICUs, as medical directors of ICUs in rural or remote areas may not be members of AMIB or affiliated with the BRICNet. We tried to mitigate this risk, by using social media to advertise the study and specifically asked colleagues to forward our invitation to medical directors of smaller ICUs in their areas and states, but it is still likely that the ICU directors who participated in the study were more academically engaged directors, in larger institutions, and stronger organizational structures, also likely to score higher on KAP. Such a selection bias may partially explain the observed association between resources and KAP score. In addition, our sample was representative of the distribution of ICUs across the territory, but there was a slight underrepresentation of the Northeast region, which should be taken into account when interpreting our results. It is also possible that the number of ICUs in the 2018 national database had changed in 2021 when we collected data. However, the total number of ICUs was used solely to calculate the percentage of ICUs in comparison to all ICUs in the country. Second, our list of resources only had one item related to ICU staffing, and therefore one of the most important resources in the ICU, human resources, is not well characterized. It also does not capture important items for managing acute respiratory failure and providing protective ventilation, such as proning or ECMO referral capability. We chose to use a previously published list of recommended resources to allow for comparisons with other studies and because the list was the result of a comprehensive examination of the literature with a focus on low- and middle-income countries., but we acknowledge that some items may be missing in the face of new evidence, such as high-flow nasal oxygen. Third, reported KAP may not reflect actual behavior at the bedside, which is what really has the potential to impact patient outcomes. In addition, we surveyed the medical directors of the ICUs, and it is possible that clinicians at the bedside have different perceptions. However, it is a useful instrument to measure a target population´s perceptions and perspectives on a given subject. The KAP survey was validated 15 years ago and therefore may be outdated in some aspects, including changing practices for managing patients with acute respiratory failure, such as light sedation, judicious use of neuromuscular blockade and use of high-flow nasal oxygen. Fourth, it is important to highlight that this is a cross-sectional study, therefore the associations found in our data do not translate into causality, In addition, both the number of resources and KAP scores were considerably high in our sample, and this ceiling effect may have impacted our power to estimate associations with accuracy. Finally, our data was collected during the COVID-19 pandemic, when ICU staff were intensively exposed to many cases of acute respiratory failure and were still suffering with physical and mental exhaustion. On the other hand, we can argue that our study provides a post-pandemic perspective, where not only technical resources were increased, as reported by most participants, but also healthcare workers became more aware of the diagnosis of ARDS and the importance of protective ventilation.

## Conclusion

This study of Brazilian ICUs revealed regional disparities in resource availability. Knowledge, attitudes, and practices related to protective mechanical ventilation were high but varied across regions, mirroring well-known disparities in access to health care and socio-economic inequalities. While overall levels of knowledge, attitudes, and practices regarding protective ventilation were generally high, significant variability was observed among participants. Although greater resource availability was associated with higher KAP scores, the magnitude of this association was modest, suggesting that factors beyond resource availability may also contribute to differences in knowledge, attitudes, and practices related to protective ventilation Our findings emphasize the need to address these regional inequalities in resource availability and promote standardized approaches to protective ventilation across ICUs in Brazil.

## Supporting information

S1 Fig DAG including relevant covariates and the association between region of the country and resources. This conceptual model using Directed acyclic graph (DAG) shows clinically relevant variables associated with resources. Arrows indicate a suspected direct causal effect of one variable on another variable. Country region is the main predictor, shown in green; Resources is the outcome, shown in blue with a vertical dash inside. Variables with a suspected impact on the outcome and also directly or indirectly associated with the main predictor, are shown in blue; additional covariates considered in the conceptual framework are shown in grey. Green arrows indicate a causal pathway. This DAG was used to evaluate the risk of bias and determine the minimal sufficient adjustment set for the multivariable analysis (according to this model, no minimal adjustment was necessary).
(DOCX)

S2 Fig DAG including relevant covariates and the association between resources and KAP. This conceptual model using Directed acyclic graph (DAG) shows clinically relevant variables associated with KAP. Arrows indicate a suspected direct causal effect of one variable on another variable. Resources is the main predictor, shown in green; KAP is the outcome, shown in blue with a vertical dash inside. Variables with a suspected impact on the outcome and also directly or indirectly associated with the main predictor, are shown in blue; additional covariates considered in the conceptual framework are shown in pink. Green arrows indicate a causal pathway, and pink arrows indicate biased pathways. This DAG was used to evaluate the risk of bias and determine the minimal sufficient adjustment set for the multivariable analysis (daily rounds and clinical research), ensuring confounding control while preserving the hypothesized causal pathways.
(DOCX)

S1 Table Number of ICUs according to region of the country. Figures are counts (percentages). Source for the national data from references 1 and 2. *Number of ICUs in the national database was obtained from Cadastro Nacional de Estabelecimentos de Saude (CNES) on August 18^th^, 2018.
(DOCX)

S2 Table Median number of resources in each state of the country. Abbreviations on the State column refer to each of the regions of Brazil. AC (Acre), AL (Alagoas), AP (Amapá), AM (Amazonas), BA (Bahia), CE (Ceará), DF (Distrito Federal), ES (Espírito Santo), GO (Goiás), MA (Maranhão), MT (Mato Grosso), MS (Mato Grosso do Sul), MG (Minas Gerais), PA (Pará), PB (Paraíba), PR (Paraná), PE (Pernambuco), PI (Piauí), RJ (Rio de Janeiro), RN (Rio Grande do Norte), RS (Rio Grande do Sul), RO (Rondônia), RR (Roraima), SC (Santa Catarina), SP (São Paulo), SE (Sergipe), TO (Tocantins).
(DOCX)

S3 Table ICU characteristics associated with the availability of resources. ICU: Intensive Care Units. * p values obtained with Linear Regression.
(DOCX)

S4 Table Characteristics of ICUs associated with the KAP score towards protective ventilation. ICU: Intensive Care Unit. * p values obtained with Linear Regression.
(DOCX)
