# Supplementary material for: The association between resource availability and knowledge, attitudes, and practice about protective ventilation among ICU directors – A nationwide cross-sectional survey
Source: PLoS One. 2026 Aug 14;21(8):e0355354. doi: 10.1371/journal.pone.0355354 (PMC13475962; doi:10.1371/journal.pone.0355354)
Supplement: Supplementary file 1 [file pone.0355354.s001.docx]

**Figure S1. DAG including relevant covariates and the association between region of the country and resources.**

**Legend:** This conceptual model using Directed acyclic graph (DAG) shows clinically relevant variables associated with resources. Arrows indicate a suspected direct causal effect of one variable on another variable. Country region is the main predictor, shown in green; Resources is the outcome, shown in blue with a vertical dash inside. Variables with a suspected impact on the outcome and also directly or indirectly associated with the main predictor, are shown in blue; additional covariates considered in the conceptual framework are shown in grey. Green arrows indicate a causal pathway. This DAG was used to evaluate the risk of bias and determine the minimal sufficient adjustment set for the multivariable analysis (according to this model, no minimal adjustment was necessary).
