# Supplementary material for: The association between resource availability and knowledge, attitudes, and practice about protective ventilation among ICU directors – A nationwide cross-sectional survey
Source: PLoS One. 2026 Aug 14;21(8):e0355354. doi: 10.1371/journal.pone.0355354 (PMC13475962; doi:10.1371/journal.pone.0355354)
Supplement: Supplementary file 2 [file pone.0355354.s002.docx]

**Figure S2. DAG including relevant covariates and the association between resources and KAP**

**Legend:** This conceptual model using Directed acyclic graph (DAG) shows clinically relevant variables associated with KAP. Arrows indicate a suspected direct causal effect of one variable on another variable. Resources is the main predictor, shown in green; KAP is the outcome, shown in blue with a vertical dash inside. Variables with a suspected impact on the outcome and also directly or indirectly associated with the main predictor, are shown in blue; additional covariates considered in the conceptual framework are shown in pink. Green arrows indicate a causal pathway, and pink arrows indicate biased pathways. This DAG was used to evaluate the risk of bias and determine the minimal sufficient adjustment set for the multivariable analysis (daily rounds and clinical research), ensuring confounding control while preserving the hypothesized causal pathways.
