# Supplementary material for: The association between resource availability and knowledge, attitudes, and practice about protective ventilation among ICU directors – A nationwide cross-sectional survey
Source: PLoS One. 2026 Aug 14;21(8):e0355354. doi: 10.1371/journal.pone.0355354 (PMC13475962; doi:10.1371/journal.pone.0355354)
Supplement: Supplementary file 3 [file pone.0355354.s003.docx]

**Table S1.** Number of ICUs according to region of the country

| **Region** | **National database (n=1806)*** | **ICU beds / 100.000 pop** | **Study sample (n=255)** |
| --- | --- | --- | --- |
| North | 100 (6%) | 8.4 | 23 (9%) |
| South | 264 (15%) | 15.2 | 45 (18%) |
| Southeast | 920 (51%) | 19.6 | 134 (52%) |
| Northeast | 363 (20%) | 10.7 | 39 (15%) |
| Midwest | 159 (9%) | 18.7 | 14 (6%) |

Footnote: figures are counts (percentages). Source for the national data from references 1 and 2. *Number of ICUs in the national database was obtained from Cadastro Nacional de Estabelecimentos de Saude (CNES) on August 18^th^, 2018
