# Supplementary material for: The association between resource availability and knowledge, attitudes, and practice about protective ventilation among ICU directors – A nationwide cross-sectional survey
Source: PLoS One. 2026 Aug 14;21(8):e0355354. doi: 10.1371/journal.pone.0355354 (PMC13475962; doi:10.1371/journal.pone.0355354)
Supplement: Supplementary file 4 [file pone.0355354.s004.docx]

**Table S2. Median number of resources in each state of the country, above and below the national median**

| **Region** | **State** | **Resources** |
| --- | --- | --- |
| North | RR | 18 |
| Northeast | PE | 30 |
| Northeast | RN | 30 |
| North | RO | 30 |
| Northeast | PB | 31 |
| North | AC | 32 |
| Midwest | GO | 32 |
| North | PA | 32 |
| Northeast | CE | 32,5 |
| Northeast | MA | 32,5 |
| North | AP | 33 |
| North | AM | 33 |
| Midwest | MS | 33 |
| South | PR | 33 |
| North | TO | 33 |
| Northeast | BA | 34 |
| Northeast | PI | 34 |
| South | SC | 34 |
| Midwest | DF | 35 |
| Southeast | ES | 35 |
| Midwest | MT | 35 |
| Southeast | MG | 35 |
| Southeast | RJ | 35 |
| South | RS | 35 |
| Southeast | SP | 35 |
| Northeast | SE | 37 |

Footnote: States shown in bleu has medina number of resources below the national median number, 34. Abbreviations on the State column refer to each of the regions of Brazil. AC (Acre), AL (Alagoas), AP (Amapá), AM (Amazonas), BA (Bahia), CE (Ceará), DF (Distrito Federal), ES (Espírito Santo), GO (Goiás), MA (Maranhão), MT (Mato Grosso), MS (Mato Grosso do Sul), MG (Minas Gerais), PA (Pará), PB (Paraíba), PR (Paraná), PE (Pernambuco), PI (Piauí), RJ (Rio de Janeiro), RN (Rio Grande do Norte), RS (Rio Grande do Sul), RO (Rondônia), RR (Roraima), SC (Santa Catarina), SP (São Paulo), SE (Sergipe), TO (Tocantins).
