# Supplementary material for: The association between resource availability and knowledge, attitudes, and practice about protective ventilation among ICU directors – A nationwide cross-sectional survey
Source: PLoS One. 2026 Aug 14;21(8):e0355354. doi: 10.1371/journal.pone.0355354 (PMC13475962; doi:10.1371/journal.pone.0355354)
Supplement: Supplementary file 5 [file pone.0355354.s005.docx]

**Table S3.** ICU characteristics associated with the availability of resources

|  | **Univariable Analysis*** | |
| --- | --- | --- |
|  | ß | ***P*** |
| Region of the country | 0,73 (0,38 – 1,09) | <0,001 |
| Hospital funding | 0,05 (-0,24 –0 ,33) | 0,74 |
| Hospital beds | 1,15 (0,56 – 1,75) | <0,001 |
| Physician 24/7 | 2,07 (-1,52 – 5,67) | 0,26 |
| ICU beds per nurse | -0,01 (-0,02 – 0,01) | 0,29 |
| Participation in research | 1,11 (0,21– 2,00) | 0,02 |
| Multidisciplinary rounds | 0,98 (0,50 – 1,46) | <0,001 |

ICU: Intensive Care Units. * p values obtained with Linear Regression
