# Supplementary material for: The association between resource availability and knowledge, attitudes, and practice about protective ventilation among ICU directors – A nationwide cross-sectional survey
Source: PLoS One. 2026 Aug 14;21(8):e0355354. doi: 10.1371/journal.pone.0355354 (PMC13475962; doi:10.1371/journal.pone.0355354)
Supplement: Supplementary file 6 [file pone.0355354.s006.docx]

**Table S4.** Characteristics of ICUs associated with the KAP score towards protective ventilation

|  | **Univariable Analysis*** | | **Multivariable Analysis*** | |
| --- | --- | --- | --- | --- |
|  | ß | ***P*** | ß | ***P*** |
| Region of the country | 0,21 (-0,81 – 1,22) | 0,69 | - | - |
| Physician 24/7 | 9,71 (-0,02 – 19,44) | 0,05 | - | - |
| Hospital funding | 0,42 (-0,35 – 1,19) | 0,29 | - | - |
| Hospital beds | -0,25 (-1,91 – 1,42) | 0,77 | - | - |
| ICU beds per nurse | 0,00 (-0,03 – 0,04) | 0,80 | - | - |
| Participation in research | 1,54 (-0,92 – 4,00) | 0,22 | 0,76 (-1,72 – 3,25) | 0,55 |
| Multidisciplinary rounds | 1,52 (0,17 – 2,86) | 0,03 | 1,07 (-0,32 – 2,46) | 0,13 |
| Resources | 0,46 (0,13 – 0,79) | 0,007 | 0,38 (0,03 – 0,72) | 0,031 |

ICU: Intensive Care Unit. * p values obtained with Linear Regression
