# Supplementary material for: The association between resource availability and knowledge, attitudes, and practice about protective ventilation among ICU directors – A nationwide cross-sectional survey
Source: PLoS One. 2026 Aug 14;21(8):e0355354. doi: 10.1371/journal.pone.0355354 (PMC13475962; doi:10.1371/journal.pone.0355354)
Supplement: Supplementary file 7 [file pone.0355354.s007.docx]

This study addresses a relevant topic with a unique focus and features a sound design and nationally representative data. However, improvements are needed in methodological transparency, in-depth analysis of the results, and the practical implications discussed. Specific comments are as follows:

Abstract:

1. The sample description lacks precision. The abstract states "255 ICUs, more than 10% the country’s ICUs," but line 120 of the manuscript indicates the sample corresponds to 12% of ICUs in the 2018 database. It is recommended to unify this description and clarify whether the temporal discrepancy between the data source (2018 database) and the survey period (2021-2022) might affect the representativeness of the findings.

2. Insufficient interpretation of the KAP score. The abstract reports a median KAP score of 83 (IQR 77-90) but does not provide the clinical or practical significance of scores on the 0-100 scale. It is suggested to supplement this with an interpretation, for example, specifying that "scores above 80 indicate a high level of knowledge, while scores of 70-80 represent a moderate level."

Introduction:

3.Lines 24-25 present the hypothesis that "lack of resources would be associated with lower KAP," but do not clearly articulate the causal relationship or underlying mechanisms between resource scarcity and KAP. Providing a theoretical rationale is necessary, as an unsupported hypothesis lacks persuasiveness and may not align with reality even if supported by data.

4.In the introduction, the authors mention regional inequalities in Brazil but do not specify the historical or socioeconomic context of these disparities (e.g., the economic development gap between the north and south). Supplementing this background would enhance the reader's understanding of the root causes of regional differences.

Data Analysis & Methods:

5. Lines 114-119 note the inability to calculate a response rate and indicate a slight underrepresentation of the Northeast region in the sample (15% vs. 20% in the national database). This may raise concerns among readers regarding potential selection bias. It is recommended to discuss the potential impact of this discrepancy on the results or to test the robustness of the findings through sensitivity analysis.

6.Lines 104-106 mention the use of Directed Acyclic Graphs (DAGs) to adjust for confounding factors, but the DAG structure or the specific covariates adjusted for are not presented in the main text or supplementary materials. It is recommended to include the DAG diagram or a list of adjusted covariates to enhance the transparency of the analysis.

7.Line 143 reports a p-value of 0.047 for the association between resources and KAP, indicating statistical significance, but the effect size (e.g., regression coefficient) is not reported. It is advised to supplement this with effect size measures (e.g., β coefficient) and discuss their clinical or practical significance. Furthermore, there is an inconsistency in the manuscript regarding whether a 5-point or 6-point Likert scale was used; the authors should clarify this point.

Results & Discussion:

8.Line 149 states that 70% of ICU directors reported an increase in resources after the pandemic, but does not analyze whether these resources are sustainable or how they might impact long-term practice. It is suggested to discuss the sustainability of post-pandemic resource increases and their potential long-term effects on protective ventilation practices.

9.The discussion should delve deeper into the causes of regional resource inequalities rather than merely describing the phenomenon. The authors could incorporate considerations of Brazil's healthcare system characteristics, policy allocation, medical infrastructure, etc., to propose targeted recommendations.

10. The discussion does not address whether the high KAP score (median 83) translates into improvements in actual clinical behavior. It is recommended to incorporate the literature on the "knowledge-practice gap" and suggest strategies to promote the translation of knowledge into practice.

Other:

The terms "protective MV" and "protective ventilation" are used interchangeably in the text; consistent terminology is recommended. Figure 1 is not described in detail within the text; it is suggested that the caption specify the resource quantity ranges corresponding to each color and that statistical analysis results of regional differences be supplemented. The discussion section lacks a statement on the limitations of this study, for instance, the inability of cross-sectional design to infer causality.
