# Supplementary material for: The association between resource availability and knowledge, attitudes, and practice about protective ventilation among ICU directors – A nationwide cross-sectional survey
Source: PLoS One. 2026 Aug 14;21(8):e0355354. doi: 10.1371/journal.pone.0355354 (PMC13475962; doi:10.1371/journal.pone.0355354)
Supplement: Supplementary file 8 [file pone.0355354.s009.docx]

We thank the dedication and time of the reviewers for critiquing our manuscript and for providing useful feedback, which we used to improve our report and it more suitable for publication at PLOS ONE.

**Editors´ comments**

Your research work is credible and addresses a key area of concern. However, there are a few uncertainties which need further clarification.
E1. The title would be better addressed as “The association between resource availability and KAP about protective vent strategies among ICU directors- A nationwide cross sectional survey”.

Thank you for the suggestion, we changed the text accordingly

2. Could 10% of ICUs be representative of the remaining 90% which did not participate?

We appreciate your point. We meant to say the geographical distribution was representative of the regions. We have edited the abstract and manuscript text to make this point more explicit

3. Line 18, grammar error- should be “ARDS patients”.

Thank you for pointing this out. We corrected it

4. Among the first 10 essential resources for ICU, defibrillator is not present. Does Brazil follow European guidelines for ICU recommendations? Is there any national guidelines?

That is a good point. The list of 10 essential resources is sponsored by the European Society of Intensive Care Medicine (ESICM) Global Intensive Care working group, and it does not include defibrillators. However, defibrillators are part of the 38 resource items, and were reported as present by 97% of respondents. Brazilian regulations requires that all ICUs have one defibrillator for each 10 beds

Reviewer #1:

This study addresses a relevant topic with a unique focus and features a sound design and nationally representative data. However, improvements are needed in methodological transparency, in-depth analysis of the results, and the practical implications discussed. Specific comments are as follows:

Abstract:
1. The sample description lacks precision. The abstract states "255 ICUs, more than 10% the country’s ICUs," but line 120 of the manuscript indicates the sample corresponds to 12% of ICUs in the 2018 database. It is recommended to unify this description and clarify whether the temporal discrepancy between the data source (2018 database) and the survey period (2021-2022) might affect the representativeness of the findings.

Thank you for your comment. We edited the abstract and substituted more than 10% with 12%, for better accuracy.

We agree that it is possible that the number of ICUs suffered slight changes from the database in 2018 to the survey period (2021-2022). However, the 2018 database was the most accurate one at the time, given that number of ICUs and ICU beds had significant and not long lasting changes during the COVID-19 pandemic. We don’t see this temporal discrepancy as affecting the representativeness of our findings because the number of ICUs from the 2018 was used solely to calculate the percentage of ICUs in comparison to all ICUs in the country, but we added a sentence about the temporal discrepancy to the limitations paragraph of our manuscript

2. Insufficient interpretation of the KAP score. The abstract reports a median KAP score of 83 (IQR 77-90) but does not provide the clinical or practical significance of scores on the 0-100 scale. It is suggested to supplement this with an interpretation, for example, specifying that "scores above 80 indicate a high level of knowledge, while scores of 70-80 represent a moderate level."

We agree that providing the readers with categories is helpful for interpretation. We added the Modified Bloom´s criteria to the methods section and a comment about the final KAP in the results section

Introduction:
3.Lines 24-25 present the hypothesis that "lack of resources would be associated with lower KAP," but do not clearly articulate the causal relationship or underlying mechanisms between resource scarcity and KAP. Providing a theoretical rationale is necessary, as an unsupported hypothesis lacks persuasiveness and may not align with reality even if supported by data.

Thank you for this comment. We had omitted it from the original version in an attempt to be concise, but we completely agree that fully articulating our hypothesis is important. We believe that lack of resources impacts staffing, access to training, availability of protocols and QI initiative, increases the workload of clinicians, and as a result impacts KAP. We added a sentence to make this association more clear

4.In the introduction, the authors mention regional inequalities in Brazil but do not specify the historical or socioeconomic context of these disparities (e.g., the economic development gap between the north and south). Supplementing this background would enhance the reader's understanding of the root causes of regional differences.

Thank you for your comment. We had omitted it from the original version in an attempt to be concise. We added a sentence to address this important perspective to the introduction.

Data Analysis & Methods:
5. Lines 114-119 note the inability to calculate a response rate and indicate a slight underrepresentation of the Northeast region in the sample (15% vs. 20% in the national database). This may raise concerns among readers regarding potential selection bias. It is recommended to discuss the potential impact of this discrepancy on the results or to test the robustness of the findings through sensitivity analysis.

We agree that this limitation needs to be very explicit. We edited the limitations paragraph to address this issue

6.Lines 104-106 mention the use of Directed Acyclic Graphs (DAGs) to adjust for confounding factors, but the DAG structure or the specific covariates adjusted for are not presented in the main text or supplementary materials. It is recommended to include the DAG diagram or a list of adjusted covariates to enhance the transparency of the analysis.

The DAG structure is presented in the supplemental material (Supplemental figures 1 and 2), and we have also mentioned some of the variables to the methods in this revised version, for more transparency.

7.Line 143 reports a p-value of 0.047 for the association between resources and KAP, indicating statistical significance, but the effect size (e.g., regression coefficient) is not reported. It is advised to supplement this with effect size measures (e.g., β coefficient) and discuss their clinical or practical significance. Furthermore, there is an inconsistency in the manuscript regarding whether a 5-point or 6-point Likert scale was used; the authors should clarify this point.

Thank you for catching this inconsistency. We could not find the exact line as mentioned by the reviewer. But in fact a p value of 0.047 was reported in the abstract, it was a typo. The correct value is 0.031. This same value was mentioned in the results section , as 0.03 – now corrected to 0.031 to be more precise – and we have now added the beta coefficient and its meaning as well.

We used a 6-point Likert scale, with scores ranging form 0 to 5 (line 78). We reviewed the manuscript to make sure this is reported as so consistently, thank you

Results & Discussion:
8.Line 149 states that 70% of ICU directors reported an increase in resources after the pandemic, but does not analyze whether these resources are sustainable or how they might impact long-term practice. It is suggested to discuss the sustainability of post-pandemic resource increases and their potential long-term effects on protective ventilation practices.

We completely agree that sustainability of these resources is crucial. Unfortunately, we did not ask about sustainability since the survey was already long and it was not one of the primary objectives of the project. We edited the sentence about it in the discussion

9.The discussion should delve deeper into the causes of regional resource inequalities rather than merely describing the phenomenon. The authors could incorporate considerations of Brazil's healthcare system characteristics, policy allocation, medical infrastructure, etc., to propose targeted recommendations.

We agree that discussion about regional disparities is important. Two paragraphs of the discussion (lines 207 to 222) address regional disparities and mention the universal health care system in Brazil, inequalities in number of ICU bed per inhabitants, and economic barriers. And we have added a deeper discussion of the disparities we found with those already known from other sources, to explore this issue more comprehensively.

10. The discussion does not address whether the high KAP score (median 83) translates into improvements in actual clinical behavior. It is recommended to incorporate the literature on the "knowledge-practice gap" and suggest strategies to promote the translation of knowledge into practice.

We agree that knowledge frequently does not translate into practice, and such an impact is one of the most difficult things to measure in real life. To address this gap, KAP scores are not limited to knowledge, they also measure reported practices and attitudes. We also worry that what is reported is not necessarily what is actually done at the bedside, and we acknowledge this limitation in the limitations section.

Other:
The terms "protective MV" and "protective ventilation" are used interchangeably in the text; consistent terminology is recommended.

Thank you for pointing this out. We corrected it and used “ventilation” throughout the manuscript

Figure 1 is not described in detail within the text; it is suggested that the caption specify the resource quantity ranges corresponding to each color and that statistical analysis results of regional differences be supplemented. The discussion section lacks a statement on the limitations of this study, for instance, the inability of cross-sectional design to infer causality.

Thank you for pointing it out, we re-wrote the figure 1 caption.

We also added a sentence to the limitations paragraph about the study design limitations to infer causality.

Reviewer #2: The manuscript presents a well-designed randomized controlled trial addressing an important clinical question regarding the prevention of postpartum depression using esketamine in women undergoing cesarean delivery. Overall, the study is technically sound, and the data largely support the conclusions; however, several areas would benefit from clarification and minor revision.

This review is not related to our manuscript, we believe that some error in the system added it to our decision letter. We will skip all comments from reviewer #2 as they are all pertaining to another submission. Thank you

From a methodological perspective, the study design is appropriate, with clearly defined inclusion and exclusion criteria, adequate randomization, and relevant outcome measures. The sample size calculation appears justified, although the assumed effect size may be somewhat optimistic and should be briefly justified. Additionally, it is not entirely clear whether an intention-to-treat analysis was performed, particularly in light of the reported loss to follow-up. Clarifying how missing data were handled would strengthen the methodological rigor.

The statistical analysis is generally appropriate and aligns with the study objectives. However, some inconsistencies in the reporting of relative risks and corresponding significance levels should be reviewed carefully to ensure coherence between the numerical results and their interpretation. Providing exact p-values where appropriate would also improve transparency.

Regarding data availability, the manuscript would benefit from a clearer and more detailed Data Availability Statement. Specifically, it should be explicitly stated whether the raw data (including individual participant-level data underlying summary statistics) are accessible, and if any restrictions apply, these should be justified in accordance with PLOS data-sharing policies.

The manuscript is generally well written and understandable, but minor grammatical and stylistic improvements are recommended to enhance clarity. Some sentences in the discussion section are slightly repetitive or could be expressed more concisely.

The discussion appropriately relates the findings to existing literature; however, some interpretations—particularly regarding the apparent preference for bolus administration—should be presented more cautiously, as the results suggest comparable efficacy between administration methods, with differences mainly in adverse events. Expanding on the clinical implications and limitations (such as the relatively short follow-up period and exclusion of patients with prenatal depression) would further strengthen the manuscript.

In conclusion, this is a valuable and clinically relevant study. Addressing the points above would improve clarity, transparency, and overall scientific robustness.

**Reviewer #3: Recommendation: Major revision**

I want to thank the editor for the opportunity to review this manuscript. The authors report a cross-sectional survey of 255 ICU directors in Brazil, capturing the availability of 38 ICU resources and 27 items measuring knowledge, attitudes, and practice (KAP) regarding protective mechanical ventilation. They report regional variation in resource availability and a statistically significant association between resource count and KAP score in adjusted analyses. The sample represents approximately 12% of Brazilian ICUs distributed across all regions, and the analysis uses pre-specified DAGs for confounder adjustment.

Overall assessment
This is a well-organized study that addresses an important question in LMIC critical care. The data collection effort is substantial, the sample is nationally representative across regions, the use of previously validated instruments is appropriate, and the DAG-based confounder adjustment is more methodologically careful than is typical for descriptive survey work. The reporting follows STROBE and the limitations section is honest about the most important constraints.

My concerns are not with the conduct of the study but with the framing and interpretation of the results. In several places the manuscript states findings more strongly than the underlying numbers support, and a few methodological points warrant further discussion. I believe these are addressable in revision and recommend major revision on the following points.

Thank you so much for your thoughtful and comprehensive review. We can truly say that your suggestions have objectively improved our manuscript.

Major points

1. The headline resource–KAP association is presented more confidently than the data warrant, and the reported p-values are inconsistent across the manuscript. The adjusted association is β = 0.38 per resource (95% CI 0.03–0.72), with the lower bound of the CI nearly at the null. The p-value is reported as 0.047 in the abstract, 0.03 in the results text, and 0.031 in Supplement Table S3. Please reconcile these values and revise the abstract and conclusion to reflect the borderline nature of the finding. The clinical magnitude should also be discussed: a β of 0.38 implies that the difference between ICUs at the 25th vs. 75th percentile of resources (33 vs. 36 resources) corresponds to roughly a 1-point change on a 0–100 KAP scale where the IQR is already 77–90. The current framing risks readers concluding that resource investment will meaningfully change KAP, which is not what these data show.

Thank you for catching the inconsistency with the p value for the association between resources and KAP. The p value of 0.047 reported in the abstract was a typo. The correct value is 0.031. This same value was mentioned in the results section, as 0.03 – now corrected to 0.031 to be more precise – and we have now added the beta coefficient and its meaning as well.

We agree with your comment, it is a significant, but small association, and we certainly do not want to imply that increasing resources will improve KAP. Not only we cannot infer causation, but also it would not be our first guess about the most suitable explanation to this association. It is much more credible that more structured ICUs, with more resources including staffing, have more training opportunities, less workload, and treatment protocols.

We have edited the manuscript throughout to acknowledge the effect size and to avoid any potential interpretation of a simple, direct, causative relationship between more resources and more KAP

2. Both primary variables exhibit substantial ceiling effects that should be addressed in the Discussion. The median resource count is 34/38 (IQR 33–36) and the median KAP score is 83/100 (IQR 77–90). Both variables are compressed at the top of their possible range, which limits the analytic power to detect resource–KAP associations and likely attenuates effect estimates. Please add a paragraph in the Limitations explicitly addressing how these ceiling effects constrain inference, and consider whether sub-analyses restricted to ICUs below the resource median (where there is more variation) would strengthen the central claim or instead attenuate it further.

We agree, and addressed it in our edited discussion and in a dedicated sentence in our limitations paragraph

3. The framing of "regional inequality" should be calibrated to the magnitudes observed. Regional medians for available resources range from 33 (North, Central West) to 35 (Southeast) out of 38 — a 2-resource gap. The p<0.001 result reflects the sample size more than the practical magnitude of the gap. The choropleth in Figure 1 uses category breaks that visually amplify a small underlying spread. I recommend (a) softening the language of regional inequality in the abstract and conclusion to reflect the modest magnitude observed in this sample, (b) reconsidering the bin breaks in Figure 1 so that the visual emphasis matches the data, and (c) explicitly contrasting the modest regional gap in resource availability within ICUs against the much larger documented regional gap in ICU bed availability per capita (referenced in the Discussion). The two are different phenomena and the current text risks conflating them.

Thank you for your comment. We agree that the regional medians are not very different in the 5 regions, given that the median was 34. However, the unequal distribution is very consistent across regions, and the map provides this information to readers. Even without knowing which states belong to each region in Brazil, any reader can see that states in the north and northwest area of the country all have less resources than states in the southeast area. In a more detailed analysis, with the median number of resources by each state, now presented in a supplemental table (Table S3, with the renumbering), we show that all of the states in the North region have medians below the national median, 34, and 4 states (two from the North region, two from the northeast region) have medians of 30 resources, while all the 4 states of the Southeast region have medians of 35 resources.

Having said that, we agree that the differences are not huge, mostly because of the ceiling effect that you mentioned. So we edited to discussion to address your comment a).

Regarding the map, point b), we don’t believe changing the bin breaks would help clarify your point a) to readers. Each bin or color includes a 2-point change in resources. Except for the outlier of 18 resources, the range goes from 30 to 37, so dividing in 5 bins felt like the most sensible approach. If each bin were to represent a 1-point change, there would be even more colors, and this could give the impression that we believe that each point change makes a difference. Dividing in less bins, let’s say, a 3-point change, would combine one state with median 37 resources with 7 states with 35 resources, and the bin going from 30 to 33 would encompass all of the states below the median, except for the outlier with 18. So instead of changing the bins, we have edited the discussion to highlight that the differences are significant but not large.

We agree with your point c) and have edited the discussion to contrast our findings with national data

4. The selection bias discussion should engage more directly with how it affects the central inferential claim. AMIB membership-based recruitment supplemented by snowball sampling on social media will systematically favor ICUs with academically engaged directors, larger institutions, and stronger organizational structures — i.e., precisely the ICUs that are also likely to score higher on KAP. The current limitations paragraph notes selection bias but does not address the possibility that the resource–KAP correlation reflects shared selection on a latent variable (institutional engagement, academic orientation, willingness to respond) rather than a causal pathway from resources to KAP. Please expand this discussion. The DAG in Figure S2 could also be revisited — "institutional engagement" or "academic affiliation" is a plausible unmeasured confounder of both resources and KAP that does not currently appear.

We agree that the sample may be biased towards more organized ICUs. We have expanded the discussion on the limitations paragraph. Your suggestion for the DAG is interesting and we agree that institutional engagement can certainly confound the association between resources and KAP. However, it is not a measurable variable, it is a complex construct that could be defined in many different ways, and we would argue that not even an approach with several questions in a questionnaire would be able to estimate it precisely. Academic affiliation can also be defined in different ways. Since the DAG was developed before any data collection or analysis and included only variables that we thought were measurable, we prefer not to modified it, and instead discuss the risk of residual confounding and selection bias in the text.

5. The 38-resource instrument is now 15 years old and omits resources directly relevant to contemporary protective ventilation. The authors note this briefly. I would ask for a more substantive paragraph in the Discussion identifying specific contemporary resources that are absent (e.g., high-flow nasal oxygen, driving pressure monitoring, esophageal manometry, ECMO referral pathways, awake prone positioning capability) and reflecting on what this means for the resource–KAP claim. The retained instrument is reasonable for cross-study comparability, but the consequence is that "resources" as measured here is only loosely related to the resources that plausibly support contemporary protective ventilation practice. Acknowledging this gap will strengthen rather than weaken the paper.

The list of resources was published in 2017, but we agree it may lack important items, especially HFNO. We also agree that ECMO referral pathways and proning capacity (awake and intubated) would be interesting to measure, and we speculate that such resources were not included in the 2017 list because they are not easily/objectively measured with a single question/item in a questionnaire, while esophageal balloons are used in very selected centers. We have edited the limitations section to strengthen limitations s of the list.

Minor points

6. The 70% figure for "increase in resources after COVID-19" is interesting but not explored. Was the magnitude of the increase quantified? Was the increase concentrated in particular regions or particular resource types? Even a brief secondary analysis would substantially strengthen the post-pandemic framing offered in the Discussion.

This is an interesting point, but unfortunately we only asked one question: did resources increase during/after the pandemic? We did not ask which resources, or how many, as the instrument was already relatively long and this was not our primary objective. Therefore, we have no insight into which resources were increased, or about sustainability. We added a sentence about sustainability as a request from the other reviewer.

7. The instrument retention from a 15-year-old survey introduces a comparability question worth noting briefly: which items in the original Dennison et al. survey are most affected by changes in clinical guidelines since 2007 (e.g., the sedation item, given the more recent ROSE trial findings the authors cite)?

We agree this is relevant and added a sentence about it in the limitations paragraph

8. The supplement title says "ICU coordinators" while the main manuscript title says "ICU directors." Please standardize.

Thank you for pointing it out, we changed the title slightly as a request of the editor, and harmonized it with the supplement

9. Supplement Table S2: the confidence interval reported for hospital beds ("1,15 (5,57 – 1,75)") appears to have a typographical error; the lower bound exceeds the point estimate. Please verify.

Thank you for catching that typo. The correct value for the lower bound of the CI was 0,557, which we now rounded to 0,56 .


The study is worth publishing, in my view, but the manuscript as submitted overstates findings that are real but modest. Calibrating the framing to match the magnitudes — and engaging more directly with selection bias and ceiling effects — would produce a stronger paper without requiring new data.
