# Supplementary material for: The association between resource availability and knowledge, attitudes, and practice about protective ventilation among ICU directors – A nationwide cross-sectional survey
Source: PLoS One. 2026 Aug 14;21(8):e0355354. doi: 10.1371/journal.pone.0355354 (PMC13475962; doi:10.1371/journal.pone.0355354)
Supplement: Peer review history

# Peer review: Decision Letter 0

**DOI:** 10.1371/journal.pone.0355354.r001

**Authors:** Shivkumar Gopalakrishnan

4 May 2026

Dear Dr. Sangia Feucht,

Please submit your revised manuscript by Jun 18 2026 11:59PM. If you will need more time than this to complete your revisions, please reply to this message or contact the journal office at plosone@plos.org. When you're ready to submit your revision, log on to https://www.editorialmanager.com/pone/ and select the 'Submissions Needing Revision' folder to locate your manuscript file.

- A letter that responds to each point raised by the academic editor and reviewer(s). You should upload this letter as a separate file labeled 'Response to Reviewers'.
- A marked-up copy of your manuscript that highlights changes made to the original version. You should upload this as a separate file labeled 'Revised Manuscript with Track Changes'.
- An unmarked version of your revised paper without tracked changes. You should upload this as a separate file labeled 'Manuscript'.

If you would like to make changes to your financial disclosure, please include your updated statement in your cover letter. Guidelines for resubmitting your figure files are available below the reviewer comments at the end of this letter.

If applicable, we recommend that you deposit your laboratory protocols in protocols.io to enhance the reproducibility of your results. Protocols.io assigns your protocol its own identifier (DOI) so that it can be cited independently in the future. For instructions see: https://journals.plos.org/plosone/s/submission-guidelines#loc-laboratory-protocols. Additionally, PLOS ONE offers an option for publishing peer-reviewed Lab Protocol articles, which describe protocols hosted on protocols.io. Read more information on sharing protocols at https://plos.org/protocols?utm_medium=editorial-email&utm_source=authorletters&utm_campaign=protocols.

As the corresponding author, your ORCID iD is verified in the submission system and will appear in the published article. PLOS supports the use of ORCID, and we encourage all coauthors to register for an ORCID iD and use it as well. Please encourage your coauthors to verify their ORCID iD within the submission system before final acceptance, as unverified ORCID iDs will not appear in the published article. *Only*  the individual author can complete the verification step; PLOS staff *cannot*  verify ORCID iDs on behalf of authors.

We look forward to receiving your revised manuscript.

Kind regards,

Shivkumar Gopalakrishnan, MD

Academic Editor

PLOS One

Journal requirements:

When submitting your revision, we need you to address these additional requirements.

1. Please ensure that your manuscript meets PLOS ONE's style requirements, including those for file naming. The PLOS ONE style templates can be found at

https://journals.plos.org/plosone/s/file?id=wjVg/PLOSOne_formatting_sample_main_body.pdf and

https://journals.plos.org/plosone/s/file?id=ba62/PLOSOne_formatting_sample_title_authors_affiliations.pdf

2. Please note that PLOS One has specific guidelines on code sharing for submissions in which author-generated code underpins the findings in the manuscript. In these cases, we expect all author-generated code to be made available without restrictions upon publication of the work. Please review our guidelines at https://journals.plos.org/plosone/s/materials-and-software-sharing#loc-sharing-code and ensure that your code is shared in a way that follows best practice and facilitates reproducibility and reuse.

If the reviewer comments include a recommendation to cite specific previously published works, please review and evaluate these publications to determine whether they are relevant and should be cited. There is no requirement to cite these works unless the editor has indicated otherwise.

Additional Editor Comments:

Dear Dr. Sangia Feucht,

Your research work is credible and addresses a key area of concern. However, there are a few uncertainties which need further clarification.

1. The title would be better addressed as “The association between resource availability and KAP about protective vent strategies among ICU directors- A nationwide cross sectional survey”.

2. Could 10% of ICUs be representative of the remaining 90% which did not participate?

3. Line 18, grammar error- should be “ARDS patients”.

4. Among the first 10 essential resources for ICU, defibrillator is not present. Does Brazil follow European guidelines for ICU recommendations? Is there any national guidelines?

Apart from the above points there are a significant number of observations by the peer reviewers which are being put up for your explanation. Please provide your inputs to these comments to enable us to arrive at a final conclusion regarding the acceptance of your article. Thank you, With regards, Dr SHIVKUMAR GOPALAKRISHNAN, MD.,

[Note: HTML markup is below. Please do not edit.]

Reviewers' comments:

Reviewer's Responses to Questions

**Comments to the Author**

1. Is the manuscript technically sound, and do the data support the conclusions?

Reviewer #1: Partly

Reviewer #2: Yes

Reviewer #3: Partly

**********

2. Has the statistical analysis been performed appropriately and rigorously?

Reviewer #1: Yes

Reviewer #2: Yes

Reviewer #3: Yes

**********

3. Have the authors made all data underlying the findings in their manuscript fully available?

The PLOS Data policy

Reviewer #1: Yes

Reviewer #2: No

Reviewer #3: Yes

**********

4. Is the manuscript presented in an intelligible fashion and written in standard English?

Reviewer #1: Yes

Reviewer #2: Yes

Reviewer #3: Yes

**********

Reviewer #1: This study addresses a relevant topic with a unique focus and features a sound design and nationally representative data. However, improvements are needed in methodological transparency, in-depth analysis of the results, and the practical implications discussed. Specific comments are as follows:

Abstract:

1. The sample description lacks precision. The abstract states "255 ICUs, more than 10% the country’s ICUs," but line 120 of the manuscript indicates the sample corresponds to 12% of ICUs in the 2018 database. It is recommended to unify this description and clarify whether the temporal discrepancy between the data source (2018 database) and the survey period (2021-2022) might affect the representativeness of the findings.

2. Insufficient interpretation of the KAP score. The abstract reports a median KAP score of 83 (IQR 77-90) but does not provide the clinical or practical significance of scores on the 0-100 scale. It is suggested to supplement this with an interpretation, for example, specifying that "scores above 80 indicate a high level of knowledge, while scores of 70-80 represent a moderate level."

Introduction:

3.Lines 24-25 present the hypothesis that "lack of resources would be associated with lower KAP," but do not clearly articulate the causal relationship or underlying mechanisms between resource scarcity and KAP. Providing a theoretical rationale is necessary, as an unsupported hypothesis lacks persuasiveness and may not align with reality even if supported by data.

4.In the introduction, the authors mention regional inequalities in Brazil but do not specify the historical or socioeconomic context of these disparities (e.g., the economic development gap between the north and south). Supplementing this background would enhance the reader's understanding of the root causes of regional differences.

Data Analysis & Methods:

5. Lines 114-119 note the inability to calculate a response rate and indicate a slight underrepresentation of the Northeast region in the sample (15% vs. 20% in the national database). This may raise concerns among readers regarding potential selection bias. It is recommended to discuss the potential impact of this discrepancy on the results or to test the robustness of the findings through sensitivity analysis.

6.Lines 104-106 mention the use of Directed Acyclic Graphs (DAGs) to adjust for confounding factors, but the DAG structure or the specific covariates adjusted for are not presented in the main text or supplementary materials. It is recommended to include the DAG diagram or a list of adjusted covariates to enhance the transparency of the analysis.

7.Line 143 reports a p-value of 0.047 for the association between resources and KAP, indicating statistical significance, but the effect size (e.g., regression coefficient) is not reported. It is advised to supplement this with effect size measures (e.g., β coefficient) and discuss their clinical or practical significance. Furthermore, there is an inconsistency in the manuscript regarding whether a 5-point or 6-point Likert scale was used; the authors should clarify this point.

Results & Discussion:

8.Line 149 states that 70% of ICU directors reported an increase in resources after the pandemic, but does not analyze whether these resources are sustainable or how they might impact long-term practice. It is suggested to discuss the sustainability of post-pandemic resource increases and their potential long-term effects on protective ventilation practices.

9.The discussion should delve deeper into the causes of regional resource inequalities rather than merely describing the phenomenon. The authors could incorporate considerations of Brazil's healthcare system characteristics, policy allocation, medical infrastructure, etc., to propose targeted recommendations.

10. The discussion does not address whether the high KAP score (median 83) translates into improvements in actual clinical behavior. It is recommended to incorporate the literature on the "knowledge-practice gap" and suggest strategies to promote the translation of knowledge into practice.

Other:

The terms "protective MV" and "protective ventilation" are used interchangeably in the text; consistent terminology is recommended. Figure 1 is not described in detail within the text; it is suggested that the caption specify the resource quantity ranges corresponding to each color and that statistical analysis results of regional differences be supplemented. The discussion section lacks a statement on the limitations of this study, for instance, the inability of cross-sectional design to infer causality.

Reviewer #2: The manuscript presents a well-designed randomized controlled trial addressing an important clinical question regarding the prevention of postpartum depression using esketamine in women undergoing cesarean delivery. Overall, the study is technically sound, and the data largely support the conclusions; however, several areas would benefit from clarification and minor revision.

From a methodological perspective, the study design is appropriate, with clearly defined inclusion and exclusion criteria, adequate randomization, and relevant outcome measures. The sample size calculation appears justified, although the assumed effect size may be somewhat optimistic and should be briefly justified. Additionally, it is not entirely clear whether an intention-to-treat analysis was performed, particularly in light of the reported loss to follow-up. Clarifying how missing data were handled would strengthen the methodological rigor.

The statistical analysis is generally appropriate and aligns with the study objectives. However, some inconsistencies in the reporting of relative risks and corresponding significance levels should be reviewed carefully to ensure coherence between the numerical results and their interpretation. Providing exact p-values where appropriate would also improve transparency.

Regarding data availability, the manuscript would benefit from a clearer and more detailed Data Availability Statement. Specifically, it should be explicitly stated whether the raw data (including individual participant-level data underlying summary statistics) are accessible, and if any restrictions apply, these should be justified in accordance with PLOS data-sharing policies.

The manuscript is generally well written and understandable, but minor grammatical and stylistic improvements are recommended to enhance clarity. Some sentences in the discussion section are slightly repetitive or could be expressed more concisely.

The discussion appropriately relates the findings to existing literature; however, some interpretations—particularly regarding the apparent preference for bolus administration—should be presented more cautiously, as the results suggest comparable efficacy between administration methods, with differences mainly in adverse events. Expanding on the clinical implications and limitations (such as the relatively short follow-up period and exclusion of patients with prenatal depression) would further strengthen the manuscript.

In conclusion, this is a valuable and clinically relevant study. Addressing the points above would improve clarity, transparency, and overall scientific robustness.

Reviewer #3: Recommendation: Major revision

I want to thank the editor for the opportunity to review this manuscript. The authors report a cross-sectional survey of 255 ICU directors in Brazil, capturing the availability of 38 ICU resources and 27 items measuring knowledge, attitudes, and practice (KAP) regarding protective mechanical ventilation. They report regional variation in resource availability and a statistically significant association between resource count and KAP score in adjusted analyses. The sample represents approximately 12% of Brazilian ICUs distributed across all regions, and the analysis uses pre-specified DAGs for confounder adjustment.

Overall assessment

This is a well-organized study that addresses an important question in LMIC critical care. The data collection effort is substantial, the sample is nationally representative across regions, the use of previously validated instruments is appropriate, and the DAG-based confounder adjustment is more methodologically careful than is typical for descriptive survey work. The reporting follows STROBE and the limitations section is honest about the most important constraints.

My concerns are not with the conduct of the study but with the framing and interpretation of the results. In several places the manuscript states findings more strongly than the underlying numbers support, and a few methodological points warrant further discussion. I believe these are addressable in revision and recommend major revision on the following points.

Major points

1. The headline resource–KAP association is presented more confidently than the data warrant, and the reported p-values are inconsistent across the manuscript. The adjusted association is β = 0.38 per resource (95% CI 0.03–0.72), with the lower bound of the CI nearly at the null. The p-value is reported as 0.047 in the abstract, 0.03 in the results text, and 0.031 in Supplement Table S3. Please reconcile these values and revise the abstract and conclusion to reflect the borderline nature of the finding. The clinical magnitude should also be discussed: a β of 0.38 implies that the difference between ICUs at the 25th vs. 75th percentile of resources (33 vs. 36 resources) corresponds to roughly a 1-point change on a 0–100 KAP scale where the IQR is already 77–90. The current framing risks readers concluding that resource investment will meaningfully change KAP, which is not what these data show.

2. Both primary variables exhibit substantial ceiling effects that should be addressed in the Discussion. The median resource count is 34/38 (IQR 33–36) and the median KAP score is 83/100 (IQR 77–90). Both variables are compressed at the top of their possible range, which limits the analytic power to detect resource–KAP associations and likely attenuates effect estimates. Please add a paragraph in the Limitations explicitly addressing how these ceiling effects constrain inference, and consider whether sub-analyses restricted to ICUs below the resource median (where there is more variation) would strengthen the central claim or instead attenuate it further.

3. The framing of "regional inequality" should be calibrated to the magnitudes observed. Regional medians for available resources range from 33 (North, Central West) to 35 (Southeast) out of 38 — a 2-resource gap. The p<0.001 result reflects the sample size more than the practical magnitude of the gap. The choropleth in Figure 1 uses category breaks that visually amplify a small underlying spread. I recommend (a) softening the language of regional inequality in the abstract and conclusion to reflect the modest magnitude observed in this sample, (b) reconsidering the bin breaks in Figure 1 so that the visual emphasis matches the data, and (c) explicitly contrasting the modest regional gap in resource availability within ICUs against the much larger documented regional gap in ICU bed availability per capita (referenced in the Discussion). The two are different phenomena and the current text risks conflating them.

4. The selection bias discussion should engage more directly with how it affects the central inferential claim. AMIB membership-based recruitment supplemented by snowball sampling on social media will systematically favor ICUs with academically engaged directors, larger institutions, and stronger organizational structures — i.e., precisely the ICUs that are also likely to score higher on KAP. The current limitations paragraph notes selection bias but does not address the possibility that the resource–KAP correlation reflects shared selection on a latent variable (institutional engagement, academic orientation, willingness to respond) rather than a causal pathway from resources to KAP. Please expand this discussion. The DAG in Figure S2 could also be revisited — "institutional engagement" or "academic affiliation" is a plausible unmeasured confounder of both resources and KAP that does not currently appear.

5. The 38-resource instrument is now 15 years old and omits resources directly relevant to contemporary protective ventilation. The authors note this briefly. I would ask for a more substantive paragraph in the Discussion identifying specific contemporary resources that are absent (e.g., high-flow nasal oxygen, driving pressure monitoring, esophageal manometry, ECMO referral pathways, awake prone positioning capability) and reflecting on what this means for the resource–KAP claim. The retained instrument is reasonable for cross-study comparability, but the consequence is that "resources" as measured here is only loosely related to the resources that plausibly support contemporary protective ventilation practice. Acknowledging this gap will strengthen rather than weaken the paper.

Minor points

6. The 70% figure for "increase in resources after COVID-19" is interesting but not explored. Was the magnitude of the increase quantified? Was the increase concentrated in particular regions or particular resource types? Even a brief secondary analysis would substantially strengthen the post-pandemic framing offered in the Discussion.

7. The instrument retention from a 15-year-old survey introduces a comparability question worth noting briefly: which items in the original Dennison et al. survey are most affected by changes in clinical guidelines since 2007 (e.g., the sedation item, given the more recent ROSE trial findings the authors cite)?

8. The supplement title says "ICU coordinators" while the main manuscript title says "ICU directors." Please standardize.

9. Supplement Table S2: the confidence interval reported for hospital beds ("1,15 (5,57 – 1,75)") appears to have a typographical error; the lower bound exceeds the point estimate. Please verify.

The study is worth publishing, in my view, but the manuscript as submitted overstates findings that are real but modest. Calibrating the framing to match the magnitudes — and engaging more directly with selection bias and ceiling effects — would produce a stronger paper without requiring new data.

**********

what does this mean?). If published, this will include your full peer review and any attached files.

If you choose “no”, your identity will remain anonymous but your review may still be made public.

**Do you want your identity to be public for this peer review?**  For information about this choice, including consent withdrawal, please see our Privacy Policy

Reviewer #1: **Yes:** Qi Jia

Reviewer #2: No

Reviewer #3: No

**********

[NOTE: If reviewer comments were submitted as an attachment file, they will be attached to this email and accessible via the submission site. Please log into your account, locate the manuscript record, and check for the action link "View Attachments". If this link does not appear, there are no attachment files.]

To ensure your figures meet our technical requirements, please review our figure guidelines: https://journals.plos.org/plosone/s/figures

You may also use PLOS’s free figure tool, NAAS, to help you prepare publication quality figures: https://journals.plos.org/plosone/s/figures#loc-tools-for-figure-preparation.

NAAS will assess whether your figures meet our technical requirements by comparing each figure against our figure specifications.

Attachment Submitted filename: Comments.docx

# Author comment: Author response to Decision Letter 1

**DOI:** 10.1371/journal.pone.0355354.r002

29 Jun 2026

our response to reviewers is attached as a document

Attachment Submitted filename: response to reviewers 18-06-26.docx

# Peer review: Decision Letter 1

**DOI:** 10.1371/journal.pone.0355354.r003

**Authors:** Shivkumar Gopalakrishnan

21 Jul 2026

The association between resource availability and knowledge, attitudes, and practice about protective ventilation among ICU directors – a nationwide cross-sectional survey

PONE-D-26-07327R1

Dear Dr. Ferreira,

We’re pleased to inform you that your manuscript has been judged scientifically suitable for publication and will be formally accepted for publication once it meets all outstanding technical requirements.

Within one week, you’ll receive an e-mail detailing the required amendments. When these have been addressed, you’ll receive a formal acceptance letter and your manuscript will be scheduled for publication.

An invoice will be generated when your article is formally accepted. Please note, if your institution has a publishing partnership with PLOS and your article meets the relevant criteria, all or part of your publication costs will be covered. Please make sure your user information is up-to-date by logging into Editorial Manager at Editorial Manager® and clicking the ‘Update My Information' link at the top of the page. For questions related to billing, please contact billing support.

If your institution or institutions have a press office, please notify them about your upcoming paper to help maximize its impact. If they’ll be preparing press materials, please inform our press team as soon as possible -- no later than 48 hours after receiving the formal acceptance. Your manuscript will remain under strict press embargo until 2 pm Eastern Time on the date of publication. For more information, please contact onepress@plos.org.

Kind regards,

Shivkumar Gopalakrishnan, MD

Academic Editor

PLOS One

Additional Editor Comments (optional):

Reviewers' comments:

Reviewer's Responses to Questions

**Comments to the Author**

Reviewer #1: All comments have been addressed

Reviewer #2: All comments have been addressed

Reviewer #3: All comments have been addressed

**********

2. Is the manuscript technically sound, and do the data support the conclusions?

Reviewer #1: Yes

Reviewer #2: Yes

Reviewer #3: Yes

**********

3. Has the statistical analysis been performed appropriately and rigorously?

Reviewer #1: Yes

Reviewer #2: Yes

Reviewer #3: Yes

**********

4. Have the authors made all data underlying the findings in their manuscript fully available?

The PLOS Data policy

Reviewer #1: Yes

Reviewer #2: Yes

Reviewer #3: Yes

**********

5. Is the manuscript presented in an intelligible fashion and written in standard English?

Reviewer #1: Yes

Reviewer #2: Yes

Reviewer #3: Yes

**********

Reviewer #1: I believe that following the author's revisions, the manuscript now demonstrates sufficient rigor and scientific validity, and I recommend that the editorial board consider it for publication.

Reviewer #2: The manuscript is scientifically sound, clearly written, and makes a valuable contribution to understanding ICU resource availability and implementation of evidence-based ventilation practices in a large middle-income country. I have no further substantive comments and believe the manuscript is suitable for publication in its current form.

Reviewer #3: The authors have thoughtfully and thoroughly addressed my comments. The revised framing now matches the magnitude of the findings, and the selection-bias and ceiling-effect limitations are appropriately acknowledged. I have no further concerns and recommend acceptance

**********

what does this mean?). If published, this will include your full peer review and any attached files.

If you choose “no”, your identity will remain anonymous but your review may still be made public.

**Do you want your identity to be public for this peer review?**  For information about this choice, including consent withdrawal, please see our Privacy Policy

Reviewer #1: **Yes:** Qi Jia

Reviewer #2: No

Reviewer #3: No

**********

# Editor report: Acceptance letter

**DOI:** 10.1371/journal.pone.0355354.r004

**Authors:** Shivkumar Gopalakrishnan

PONE-D-26-07327R1

PLOS One

Dear Dr. Ferreira,

I'm pleased to inform you that your manuscript has been deemed suitable for publication in PLOS One. Congratulations! Your manuscript is now being handed over to our production team.

At this stage, our production department will prepare your paper for publication. This includes ensuring the following:

* All references, tables, and figures are properly cited

* All relevant supporting information is included in the manuscript submission,

* There are no issues that prevent the paper from being properly typeset

You will receive further instructions from the production team, including instructions on how to review your proof when it is ready. Please keep in mind that we are working through a large volume of accepted articles, so please give us a few days to review your paper and let you know the next and final steps.

Lastly, if your institution or institutions have a press office, please let them know about your upcoming paper now to help maximize its impact. If they'll be preparing press materials, please inform our press team within the next 48 hours. Your manuscript will remain under strict press embargo until 2 pm Eastern Time on the date of publication. For more information, please contact onepress@plos.org.

You will receive an invoice from PLOS for your publication fee after your manuscript has reached the completed accept phase. If you receive an email requesting payment before acceptance or for any other service, this may be a phishing scheme. Learn how to identify phishing emails and protect your accounts at https://explore.plos.org/phishing.

If we can help with anything else, please email us at customercare@plos.org.

Thank you for submitting your work to PLOS ONE and supporting open access.

Kind regards,

PLOS ONE Editorial Office Staff

on behalf of

Dr. Shivkumar Gopalakrishnan

Academic Editor

PLOS One
